# Repigmentation Competence in Vitiligo: Integrating Immune, Regulatory, Regenerative, and Microenvironmental Axes

**DOI:** 10.3390/jpm16080418

**Published:** 2026-08-06

**Authors:** Maria Efenesia Baffa, Roberto Maglie, Stefano Colabrese, Carlo Pipitò, Vincenzina Rubino, Sasha Visinoni, Lucrezia Cerchiai, Marzia Caproni, Emiliano Antiga

**Affiliations:** 1 Department of Health Sciences, Section of Dermatology, University of Florence, 50125 Florence, Italy; mariaefenesia.baffa@unifi.it (M.E.B.); stefano.colabrese@unifi.it (S.C.); carlo.pipito@unifi.it (C.P.); vincenzina.rubino@unifi.it (V.R.); sasha.visinoni@unifi.it (S.V.); lucrezia.cerchiai@unifi.it (L.C.); emiliano.antiga@unifi.it (E.A.); 2 Rare Disease Dermatological Unit, Department of Health Sciences, Section of Dermatology, University of Florence, P. Palagi Hospital, 50125 Florence, Italy; marzia.caproni@unifi.it

**Keywords:** vitiligo, treatment, autoimmunity, immune regulation, microenvironment, repigmentation competence

## Abstract

Vitiligo is an autoimmune depigmenting disorder characterized by marked heterogeneity in therapeutic response, both between patients and among lesions within the same individual. While current therapies primarily target interferon-γ (IFN-γ)-driven inflammation, clinical outcomes remain variable and frequently incomplete, suggesting that additional lesion-specific biological factors contribute to repigmentation potential. In this narrative review, we propose the concept of *repigmentation competence* to describe the capacity of an individual lesion to achieve clinically meaningful repigmentation under therapy. We hypothesize that this competence emerges from the interaction of four interconnected biological axes: (i) cytokine network and immune memory, (ii) local immune regulation, (iii) regenerative capacity, and (iv) microenvironmental permissiveness. A targeted search of PubMed/MEDLINE and ClinicalTrials.gov up to March 2026 was conducted to identify translational studies, mechanistic models, and clinical trials relevant to these pathways. Evidence was synthesized thematically with emphasis on cytokine-mediated mechanisms and their interaction with regenerative and tissue-context processes. The IFN-γ/CXCL9/CXCL10 axis and tissue-resident memory T cells (TRM) represent the most clinically validated drivers of disease persistence, as demonstrated by the therapeutic efficacy of JAK inhibitors, although immune suppression alone often fails to achieve complete or durable repigmentation. In contrast, regulatory pathways involving PD-1/PD-L1 signaling, regulatory T cells (Tregs), IL-10, TGF-β, and IL-2–based strategies remain biologically compelling but only partially translated into effective therapies. Regenerative capacity has also emerged as an important determinant of treatment response, with growing evidence supporting the role of melanocyte stem cell niches, follicular regeneration, and Wnt/β-catenin signaling. Accordingly, regenerative approaches such as non-cultured epidermal cell suspension (NCES) are increasingly being integrated into combination therapeutic strategies. The lesional microenvironment remains the least therapeutically developed axis despite growing experimental evidence supporting its importance in melanocyte survival and migration. Collectively, these observations suggest that the variable efficacy of current therapies may reflect different combinations of lesion-specific biological constraints. The emerging benefit of combination strategies may therefore derive not simply from additive effects, but from the simultaneous engagement of multiple axes involved in repigmentation competence. Our review supports a shift from a predominantly drug-centered model toward a lesion-oriented framework integrating immune, regenerative, regulatory, and microenvironmental determinants of response.

## 1. Introduction

Vitiligo is an autoimmune skin disease characterized by the selective loss of melanocytes and the presence of non-scaling, irregular, achromic patches. Vitiligo affects approximately 0.5% to 2% of the global population and, despite its limited physical morbidity, carries a substantial burden largely driven by its visibility, chronicity, and unpredictable clinical course [1].

Over the last decade, advances in immunology have established a central role for type 1 immune responses driven by interferon-γ (IFN-γ). Autoreactive CD8+ T cells infiltrate the skin and mediate melanocyte destruction, while IFN-γ stimulates keratinocytes to produce chemokines such as CXCL9 and CXCL10, which in turn recruit additional CXCR3-expressing T cells, reinforcing a self-sustaining inflammatory loop [2,3]. This mechanistic framework has enabled the development of targeted therapies, particularly Janus kinase (JAK) inhibitors, which act on IFN-γ signaling and have demonstrated clinically meaningful repigmentation [4].

A striking feature of vitiligo is the heterogeneity of treatment response, not only between patients but also between lesions within the same individual. While anatomical location has traditionally been used to explain differences in response, facial lesions being more responsive than acral areas, clinical experience consistently reveals that even lesions located within the same anatomical region may behave differently under identical therapeutic conditions.

This variability suggests that therapeutic response is shaped by lesion-level biological heterogeneity, which is not fully captured by conventional patient-level predictors.

To conceptualize this lesion-specific variability, we propose the term *repigmentation competence*, defined as the capacity of an individual lesion to achieve clinically meaningful repigmentation under therapy. The aim of this narrative review is to examine the biological determinants of repigmentation competence by integrating current evidence across four interconnected axes: (i) cytokine network and immune memory, (ii) local immune regulation, (iii) regenerative capacity, and (iv) microenvironmental permissiveness. Through this framework, we seek to provide a lesion-oriented perspective that may help explain heterogeneous therapeutic responses and support the rationale for future multi-target treatment strategies.

## 2. Materials and Methods

This narrative review was conducted through a targeted search of PubMed/MEDLINE up to March 2026, complemented by a structured review of ClinicalTrials.gov for interventional studies relevant to vitiligo.

Search terms included combinations of “vitiligo,” “IFN-γ,” “CXCL9,” “CXCL10,” “IL-15,” “JAK inhibitors,” “tissue-resident memory T cells,” “regulatory T cells,” “PD-1,” “melanocyte stem cells,” “hair follicle niche,” “Wnt signaling,” “repigmentation,” “oxidative stress,” “adhesion,” “E-cadherin,” “microenvironment,” and “immune regulation.”

Relevant preclinical studies, translational human investigations, and clinical trials were selected based on their contribution to the understanding of mechanisms potentially influencing repigmentation. Evidence was synthesized narratively and organized according to a conceptual framework centered on lesion-level determinants of therapeutic response. Attention was given to the translational relevance of the identified pathways, their therapeutic targetability, and their implications for future clinical research and therapeutic development.

### 2.1. Axis 1–Cytokine Network and Immune Memory

The occurrence and persistence of vitiligo lesions are sustained by a cytokine network that supports both effector activity and long-term immune memory. Genetic studies independently support the centrality of this immune-effector axis, with genome-wide association analyses consistently identifying strong susceptibility signals within the HLA class I and II regions and genes involved in adaptive immune activation [5]. Central to this network is IFN-γ, which orchestrates the recruitment and activation of autoreactive T cells through induction of CXCL9 and CXCL10 in keratinocytes. Rashighi et al. demonstrated that CXCL10 is not merely a biomarker but a functional driver of disease, as its neutralization leads to reversal of depigmentation in murine models. This finding established the IFN-γ/CXCL10 axis as a critical component of disease maintenance and a relevant therapeutic target [2].

The clinical efficacy of JAK inhibitors provides strong translational validation of this pathway. Mechanistically, IFN-γ signals through the JAK/STAT pathway, inducing CXCL9 and CXCL10 expression in keratinocytes. Accordingly, JAK inhibition interrupts IFN-γ signaling and limits the CXCL10-dependent recruitment of autoreactive T cells [2,3]. Across multiple agents targeting different components of the JAK/STAT cascade, clinically meaningful repigmentation has been consistently observed [4,6,7,8,9,10,11,12,13,14,15,16,17] (Table 1). Notably, topical ruxolitinib, the first approved agent for vitiligo therapy, has demonstrated robust efficacy in phase 3 trials [4] as well as in real-world studies [18]. However, the variability in response indicates that suppression of this axis alone does not uniformly restore pigmentation.

A key factor underlying this limitation may be the persistence of tissue-resident memory T cells (TRM), which remain in lesional skin and can reactivate inflammation. IL-15 plays an important role in maintaining these cells, although additional survival signals within the tissue microenvironment are likely to contribute. In a landmark study, Richmond et al. demonstrated that TRM in vitiligo depend on IL-15 signaling for survival and function, and that blockade of this pathway leads to durable repigmentation in animal models. Importantly, the study revealed distinct temporal effects: early inhibition reduces cytokine production, whereas prolonged blockade leads to depletion of TRM [19].

However, translation into clinical benefit has been less striking than anticipated. The trial targeting IL-15 (NCT04338581) suggests that while modulation of immune memory is achievable, this does not necessarily translate into robust repigmentation [34]. This discrepancy likely reflects the fact that IL-15 blockade modulates persistence without fully eliminating the pathogenic cellular compartment. Consistently, JAK inhibition, while clinically effective, does not eradicate TRM [20], which remains capable of sustaining local disease activity.

All these findings indicate that the IFN-γ/IL-15 cytokine network represents a central effector axis of disease activity. However, even when effectively targeted, modulation of this immune circuitry does not uniformly translate into repigmentation, suggesting that additional lesion-specific factors contribute to the overall repigmentation competence of individual lesions. This concept is also supported by genetic studies, as genome-wide association analyses consistently identify susceptibility loci involved in antigen presentation and T-cell activation, including HLA class I/II genes, *PTPN22*, and *GZMB*, highlighting the central role of adaptive immune activation in vitiligo pathogenesis [5].

### 2.2. Axis 2–Regulatory Cytokines and Local Immune Regulation

While IFN-γ–driven inflammation is necessary for disease progression, its impact is modulated by local regulatory mechanisms that determine whether autoreactive T cells remain pathogenic or are functionally restrained. Single-cell analyses indicate that clinically unaffected skin in vitiligo is not immunologically inert, as it may already contain autoreactive CD8+ T cells with transcriptional features similar to those found in lesional skin. However, these cells exhibit increased expression of inhibitory receptors such as PD-1 and coexist with higher levels of regulatory T cells (Tregs), suggesting a state of controlled activation [35]. These observations imply that progression from normal-appearing skin to active depigmentation depends not only on effector pathways but also on the failure of local immune regulation.

A growing body of evidence supports the role of Tregs in this process. Earlier mechanistic studies implicated Treg deficiency or dysfunction in vitiligo [21,22,36], and a recent systematic review and meta-analysis confirmed reduced Treg numbers, impaired suppressive function toward CD8+ T cells, lower IL-10 levels, and increased levels of Th17-related cytokines [37]. Together, these findings suggest that defective immune regulation may represent an important component of disease biology rather than a mere consequence of inflammation.

Within this framework, the PD-1/PD-L1 axis has emerged as a particularly attractive regulatory pathway. Clinical observations from oncology demonstrate that disruption of this checkpoint can precipitate vitiligo-like depigmentation [38], whereas experimental activation of PD-1/PD-L1 signaling promotes immune tolerance and limits melanocyte-reactive responses [23]. Recent evidence further suggests that checkpoint dysfunction in vitiligo may involve not only autoreactive T cells but also the target tissue itself. While IFN-γ normally induces PD-L1 expression in healthy melanocytes, keratinocytes, and fibroblasts as part of a physiological negative feedback mechanism, melanocytes derived from patients with vitiligo appear unable to appropriately upregulate PD-L1 in response to IFN-γ stimulation. Consequently, the same cytokine driving melanocyte destruction may fail to trigger the checkpoint signals that normally limit tissue damage, creating a self-perpetuating cycle of inflammation and melanocyte loss [24,39].

Other regulatory mediators, including IL-10 and TGF-β, are also likely to contribute to melanocyte tolerance, although their precise roles in human vitiligo remain incompletely defined.

From a therapeutic perspective, selective Treg expansion through IL-2–based approaches represents one of the most biologically appealing strategies. IL-2 signaling is fundamental for Treg development, homeostasis, and suppressive function, and low-dose or Treg-biased IL-2 biologics can preferentially expand Tregs in vivo [40]. Consistent with the potential biological relevance of this pathway, genetic studies have identified susceptibility loci involving *IL2RA*, while associations between *FOXP3* promoter polymorphisms and vitiligo susceptibility further support the contribution of pathways regulating Treg development and immune tolerance [5,6,7,8,9,10,11,12,13,14,15,16,17,18,19,20,21,22,23,24,34,35,36,37,38,39,40,41]. However, experience from other autoimmune diseases suggests that robust Treg expansion does not always translate into meaningful clinical benefit [42,43]. In vitiligo, a phase 2a trial of the Treg-selective IL-2 mutein MK-6194/PT101 was initiated but terminated for business reasons before efficacy data became available [44].

Beyond Treg-directed approaches, restoration of checkpoint signaling has emerged as a potential therapeutic avenue. In a murine model of vitiligo, PD-L1 fusion protein treatment induced approximately 60% recovery of lost pigmentation while increasing Treg abundance and reducing melanocyte-reactive effector T cells [23]. Clinical interest in checkpoint restoration is also reflected by exploratory studies investigating cytotoxic T-lymphocyte-associated protein 4 (CTLA-4) agonism with abatacept [45], although its efficacy in vitiligo remains unknown. More recently, melanocyte-targeted bispecific PD-1 agonists have been proposed to restore local immune tolerance directly at sites of autoimmune attack while minimizing systemic immunosuppression [46].

Although these approaches remain experimental, they support the biological relevance of local immune regulation as a distinct axis of vitiligo pathogenesis.

### 2.3. Axis 3–Regenerative Capacity

Repigmentation in vitiligo depends not only on suppressing melanocyte-directed inflammation, but also on the presence of a functional melanocyte reservoir capable of regenerating epidermal pigmentation.

This capacity extends beyond the mere presence of residual pigment cells and reflects the integrity of a functional unit composed of melanocyte stem cells and their follicular niche, which together provide the cellular substrate for epidermal recolonization.

Classical histological studies demonstrated that repigmentation typically originates from the hair follicle and proceeds centrifugally, supporting the concept that inactive melanocytes in the outer root sheath constitute a reservoir for epidermal recolonization [25]. In vitiligo, while epidermal melanocytes are lost, follicular melanocytes may persist and serve as a source of repigmentation under treatment.

This reservoir concept was subsequently refined by work identifying melanocyte stem cells in the bulge region of the hair follicle and demonstrating that these cells are slow-cycling, self-maintaining, and capable of regenerating differentiated melanocytes during the next growth cycle [26]. In other words, the follicle is not simply an anatomic shelter, but a functional cell niche.

More recent data indicate that therapeutic repigmentation requires active engagement of this niche. In NB-UVB-treated vitiligo skin, melanocyte precursors within the bulge show upregulation of *GLI1* and activation of β-catenin–dependent pathways, together with gene programs related to proliferation, migration, adhesion, and stemness [27]. This suggests that repigmentation is an actively regulated process rather than a passive consequence of immune suppression. Within this context, Wnt/β-catenin signaling appears to play a role in promoting melanocyte stem cell activation and differentiation and has been proposed as a potential therapeutic target to enhance regenerative responses [28].

At the same time, this regenerative unit is vulnerable to inflammatory disruption. Experimental single-cell data suggest that melanocyte stem cells and mature melanocytes may undergo distinct stress-related fates, including pyroptosis, ferroptosis, oxidative stress, and mitochondrial dysfunction. The same study also showed that the hair follicle niche can acquire an inflammatory profile, with increased expression of IL-1, IL-6, IL-15, CCL2, CXCL12 and IFN-γ pathway genes in adjacent follicular compartments, raising the possibility that impaired regeneration may reflect not only precursor depletion but also dysfunction of the surrounding niche [29].

From a therapeutic perspective, this point is highly relevant. If a lesion has lost its effective follicular reservoir—as may occur in leukotrichia-rich lesions, long-standing disease, or glabrous sites—immune control alone may be insufficient because the lesion lacks the cellular substrate for repigmentation. This concept is supported by clinical observations that such lesions are consistently less responsive to medical therapy, even under optimal immunomodulation.

This limitation has led to the development of regenerative and cell-based approaches aimed at restoring melanocyte availability rather than simply modulating immune activity. Autologous non-cultured epidermal cell suspension (NCES) has demonstrated efficacy in stable vitiligo, including in within-patient comparative designs, with durable repigmentation in a subset of treated lesions [30], although outcomes remain dependent on disease stability, anatomical location, and procedural factors.

In this regard, the ongoing trial of upadacitinib following NCES (NCT06454461) represents a combined strategy of regenerative replacement with post-procedural immune control, reflecting the concept that successful repigmentation after cell transfer may still depend on maintaining an immunologically permissive environment [47].

Overall, these findings indicate that regenerative capacity reflects the interplay between melanocyte precursor availability, niche integrity, and local signaling pathways. Within a lesion-based framework, this axis may represent a critical determinant of repigmentation competence, as lesions lacking a functional regenerative reservoir are unlikely to respond to immunomodulatory strategies alone. From a therapeutic standpoint, current approaches appear to operate along two complementary directions: reactivating a residual niche or compensating for an insufficient reservoir through cellular replacement.

### 2.4. Axis 4–Microenvironmental Permissiveness

While individual cytokines and cellular compartments provide essential mechanistic insights, repigmentation ultimately occurs within a complex tissue context in which immune signals, structural components, and metabolic factors converge. In this sense, the lesional microenvironment can be conceptualized as an extended tissue niche integrating keratinocytes, fibroblasts, extracellular matrix components, and local cytokine networks. Importantly, this altered microenvironment may extend, at least in part, to clinically normal-appearing skin, which is increasingly recognized as biologically distinct from healthy skin [48].

Within this integrated system, keratinocytes play an important role as both amplifiers of immune signaling and regulators of melanocyte homeostasis. Beyond their immunological functions, keratinocytes regulate melanocyte survival, positioning, and differentiation through direct cell–cell interactions and paracrine signaling, consistent with prior models emphasizing epidermal cross-talk in melanocyte regeneration [49].

Melanocyte anchorage to the basal epidermis is primarily mediated by epithelial-cadherin (E-cadherin), which ensures stable interaction with keratinocytes. In vitiligo, altered expression and distribution of E-cadherin have been documented even in non-lesional skin, suggesting that defective adhesion may precede overt depigmentation [31], although its causal role in melanocyte loss remains to be fully established. This concept aligns with the theory of melanocytorrhagy, in which impaired adhesion renders melanocytes susceptible to detachment and loss under conditions of mechanical or inflammatory stress [32]. Consistent with this concept, genetic association studies have identified susceptibility variants in the adhesion-related genes *CDH1* and Discoidin Domain Receptor Tyrosine kinase 1 (*DDR1*), further supporting the contribution of impaired melanocyte adhesion to vitiligo pathogenesis [50].

Cytokine signaling and oxidative stress appear to directly modulate these adhesion pathways. IFN-γ and other pro-inflammatory mediators can disrupt keratinocyte–melanocyte interactions, while oxidative stress, well documented in vitiligo, can further destabilize cytoskeletal organization and extracellular matrix integrity [51]. These findings suggest that melanocyte loss may result not only from immune-mediated cytotoxicity, but also from a failure of retention within a structurally and metabolically permissive niche.

Beyond E-cadherin, additional adhesion and matrix-related components contribute to melanocyte stability and migration. Integrins, laminins, and extracellular matrix proteins such as tenascin have been implicated in both anchoring melanocytes to the basement membrane and enabling their migration during repigmentation. In parallel, keratinocyte- and fibroblast-derived trophic factors, including stem cell factor (SCF), hepatocyte growth factor (HGF), and endothelin-1 (EDN1), support melanocyte survival and function, suggesting that the microenvironment can be permissive, neutral, or actively hostile depending on its biochemical composition [52].

From a therapeutic perspective, this axis remains markedly underdeveloped compared to immune-targeted strategies. Although no approved treatments directly target adhesion pathways or microenvironmental stability in vitiligo, several interventions may exert indirect effects. Phototherapy, particularly narrowband UVB, has been shown to improve keratinocyte function and modulate the local environment, potentially enhancing melanocyte retention and migration [27].

More recently, translational models have begun to explore whether these pathways can be directly modulated. In an induced pluripotent stem cell (iPSC)-based vitiligo model, inhibition of Rho-associated protein kinase (ROCK) restored melanocyte–keratinocyte crosstalk under IFN-γ exposure, upregulating adhesion molecules such as E-cadherin and DDR1, as well as trophic factors including basic fibroblast growth factor and endothelin-1. Functionally, this resulted in improved melanocyte survival and recovery of dendritic architecture, suggesting that structural instability within the microenvironment is not merely a downstream consequence of inflammation but may represent a pharmacologically targetable process [33].

Among emerging approaches, melanocortin-based therapies represent one of the few strategies aimed at enhancing melanocyte function rather than suppressing immune activity. Afamelanotide, an α-melanocyte-stimulating hormone analogue, has shown enhanced repigmentation when combined with NB-UVB and continues to be evaluated in advanced clinical settings. By promoting melanocyte survival and melanogenesis through melanocortin 1 receptor (MC1R) signaling, this strategy may partially compensate for an unfavorable microenvironment [53]. However, this approach remains intrinsically limited, as it enhances the function of existing melanocytes without directly addressing deficits in the melanocyte stem cell reservoir [54].

The lesional microenvironment seems to function as an integrative niche in which immune activity, structural integrity, and regenerative processes converge. Within the framework of repigmentation competence, this axis may act by modulating the ability of melanocytes to persist, migrate, and function within the tissue, even when immune and regenerative pathways are adequately targeted. Notably, most microenvironmental determinants remain indirectly or experimentally targeted, highlighting a gap between biological understanding and therapeutic development.

## 3. Conclusions and Future Directions

### 3.1. Repigmentation Competence as an Emergent Lesion Property

Repigmentation in vitiligo appears to be the result of multiple interacting biological processes that operate at the level of individual lesions. The variability in therapeutic response observed in clinical practice, both between patients and across lesions in the same patient, suggests that acting on a single pathway may not be sufficient to achieve meaningful clinical outcomes.

**Figure 1:**
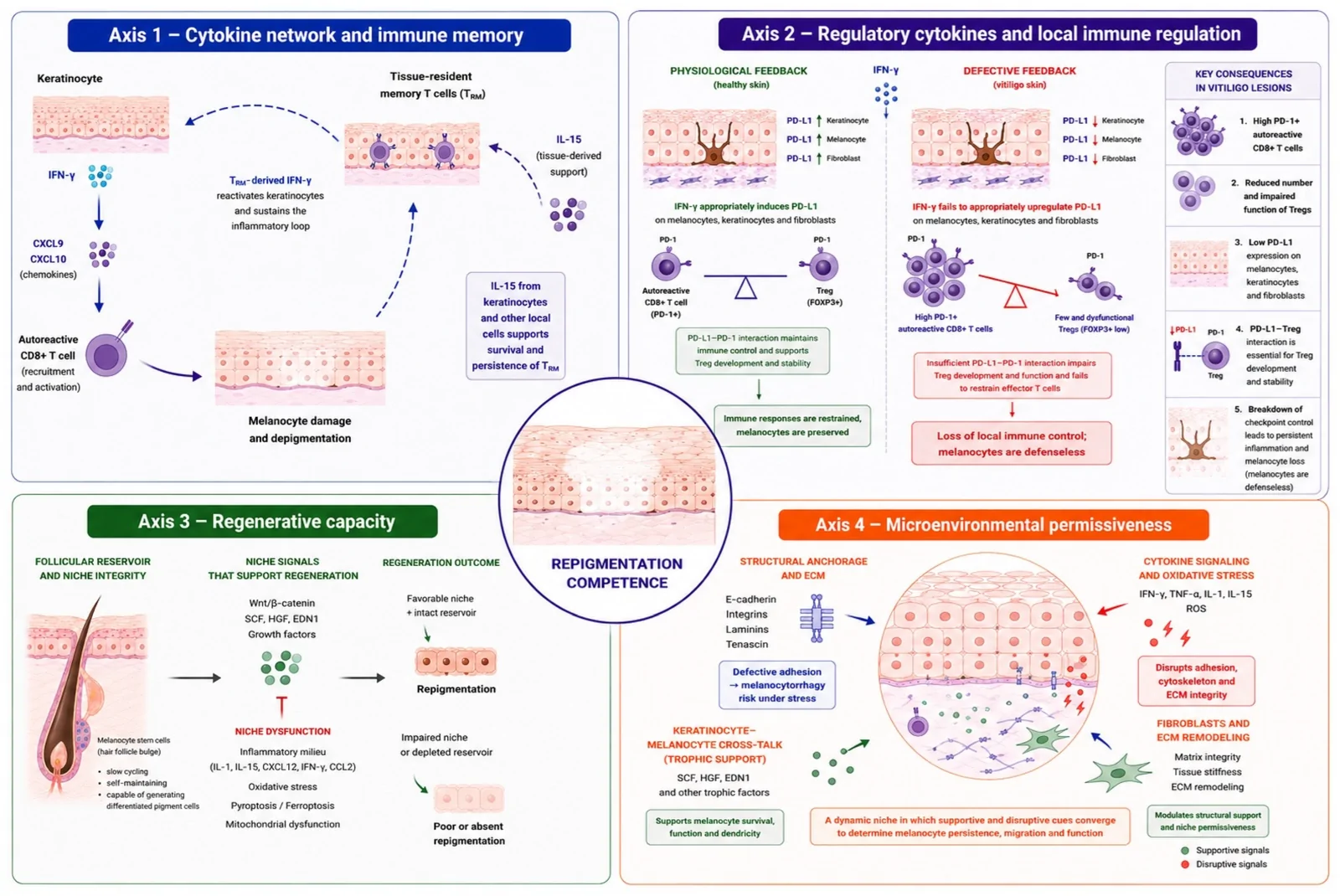
Repigmentation competence is conceptualized as an emergent lesion-level property resulting from the interaction of four interconnected biological axes. Axis 1 (Cytokine network and immune memory) illustrates the IFN-γ/CXCL9/CXCL10 pathway and the contribution of tissue-resident memory T cells (TRM), sustained in part by IL-15, to disease persistence and melanocyte loss. Axis 2 (Regulatory cytokines and local immune regulation) highlights mechanisms of immune restraint, including regulatory T cells (Tregs), PD-1/PD-L1 signaling, and regulatory mediators such as IL-10 and TGF-β, which may modulate autoreactive T-cell activity and maintain melanocyte tolerance. Axis 3 (Regenerative capacity) represents the follicular melanocyte stem-cell reservoir, niche integrity, and regenerative pathways involved in melanocyte activation, migration, and differentiation, as well as factors associated with niche dysfunction. Axis 4 (Microenvironmental permissiveness) depicts the tissue context in which repigmentation occurs, including cell adhesion pathways, keratinocyte–melanocyte crosstalk, extracellular matrix remodeling, oxidative stress, and stromal support. The central depigmented lesion represents the integrated outcome of these processes, referred to as *repigmentation competence*. The model is intended as a conceptual framework to explain lesion-specific variability in therapeutic response and to provide a rationale for multi-axis combination strategies.

Rather than acting as independent biological modules, these axes continuously influence one another. Persistent inflammatory signaling may impair local immune regulation, alter the regenerative potential of the follicular niche, and remodel the tissue microenvironment, whereas changes in the regenerative compartment or tissue microenvironment may in turn modulate immune activation and therapeutic responsiveness (Figure 1).

Consequently, repigmentation competence should be viewed as an emergent property of the lesion resulting from the dynamic interplay among these interconnected biological processes, rather than from the activity of any single axis alone. From this perspective, therapeutic efficacy may depend on the extent to which these lesion-specific constraints can be simultaneously modulated. These observations suggest that repigmentation competence should be regarded as an integrated biological property of individual lesions rather than the consequence of any single pathogenic mechanism.

### 3.2. Clinical Implications

Although current therapies primarily target the IFN-γ-driven immune axis, their effects remain variable and often incomplete. This observation suggests that, in many lesions, additional biological limitations, such as impaired regenerative capacity or an unfavorable microenvironment, may restrict the ability to achieve sustained repigmentation (Table 2).

From a clinical perspective, this framework suggests that treatment failure should not necessarily be interpreted as inadequate immune suppression alone. Rather, different lesions may fail to repigment because distinct biological constraints predominate. Although this concept is not yet sufficiently validated to guide routine treatment selection, it provides a biological rationale for interpreting heterogeneous therapeutic responses and supports the future development of lesion-oriented therapeutic algorithms.

Several recent translational studies indirectly support the existence of biologically distinct lesional states associated with differential therapeutic behavior. In a biomarker analysis of ritlecitinib-treated vitiligo lesions, active lesions displayed higher expression of inflammatory markers such as *IFNG*, *CCL5*, CXCL9, PD-L1, CD103, and T-cell infiltrates, whereas lesions with more stable behavior showed higher HO-1 levels and demonstrated earlier repigmentation under treatment [33]. Notably, the authors observed that stable lesions appeared capable of repigmenting earlier, whereas active lesions required prior inflammatory stabilization before clinically meaningful repigmentation could occur [55]. These findings also illustrate how lesion-level biomarker profiling may provide an objective framework for future evaluation of repigmentation competence, particularly when integrated with histopathological and immunohistochemical assessment of the biological axes proposed in this review. Although these findings do not directly validate a multi-axis model, they support the notion that lesions may exist in biologically distinct states that influence their responsiveness to therapy.

Phototherapy offers a useful, albeit imperfect, illustration of this complexity. While its clinical efficacy is well established, treatment response is heterogeneous. Mechanistic studies indicate that NB-UVB extends beyond immune modulation, promoting activation of melanocyte precursors and modulating pathways involved in proliferation, migration, adhesion, and Wnt/β-catenin signaling within the follicular niche [27]. This broader biological activity suggests that engagement of multiple axes may contribute to therapeutic success, although it does not uniformly overcome all lesion-specific constraints.

### 3.3. Future Directions

This rationale is increasingly reflected in the development of combination strategies. The addition of NB-UVB to JAK inhibition, including topical ruxolitinib and systemic agents such as baricitinib, has been associated with improved repigmentation compared with monotherapy in both clinical studies and ongoing trials [7,10,57]. Similarly, combined approaches integrating immune modulation with regenerative interventions, such as upadacitinib following non-cultured epidermal cell suspension transplantation (NCT06454461), aim to address both IFN-γ-induced inflammation and melanocyte availability [47]. Other strategies, including melanocortin analogues combined with phototherapy (NCT04525157), further support the concept that targeting complementary biological processes may enhance treatment response [53,56].

Taken together, these observations suggest that future therapeutic strategies in vitiligo may benefit from a shift from a predominantly drug-centered paradigm to a more lesion-oriented approach, in which treatments are tailored to the dominant biological constraints within each lesion. Such an approach will likely require consideration not only of the predominant biological axis in a given lesion but also of the reciprocal interactions among these processes, which together determine the overall capacity for repigmentation.

However, this framework remains largely conceptual and should not be interpreted as a validated biological classification system. Current evidence remains fragmented, largely indirect, and frequently derived from heterogeneous sources—including in vitro studies, animal models, translational datasets, and clinical investigations—rather than prospective lesion-level studies specifically designed to test multi-axis therapeutic models. Further research integrating mechanistic biomarkers, longitudinal lesion profiling, and interventional clinical trial design will be required to determine whether this conceptual framework can be translated into improved and more consistent clinical outcomes. Such studies may ultimately enable lesion-level patient stratification, biomarker-guided therapeutic selection, and the rational design of personalized combination therapies aimed at maximizing repigmentation. This approach may guide the development of future therapies targeting the specific biological processes that limit repigmentation within individual lesions.

## Figures and Tables

**Table 1:** Representative primary experimental and translational evidence supporting the biological axes of repigmentation competence in vitiligo.

Axis	Study	Study Design/Model	Representative Experimental Evidence	Relevance to Repigmentation Competence	Ref
Axis 1. Cytokine network and immune memory	Rashighi et al.	Murine vitiligo model; chemokine analysis and antibody blockade	CXCL10 was increased in affected skin. CXCL10 neutralization reversed established depigmentation, whereas CXCL9 blockade did not show comparable efficacy.	Establishes CXCL10 as a functional driver of disease maintenance and a therapeutic target.	[2]
	Richmond et al.	Human samples and murine model; anti-CD122 intervention	Lesional T cells display TRM, which express the CD122 subunit of the IL-15 receptor. Targeting IL15 signaling with anti-CD122 reverses vitiligo in mice.	Shows that IL-15 sustains both TRM effector function and maintenance.	[19]
	Azzolino et al.	Murine model treated with JAK inhibitors	Tofacitinib and ruxolitinib prevented progression and reversed depigmentation without eliminating epidermal TRM.	JAK inhibitors in mice can restore pigmentation while leaving a TRM reservoir.	[20]
Axis 2. Regulatory cytokines and local immune regulation	Klarquist et al.	Human biopsies immunohistochemistry, blood phenotyping, Treg suppression and chemotaxis assays	Cutaneous Tregs were reduced despite preserved abundance and activity of circulating Tregs; reduced skin CCL22 may be associated with impaired Treg skin homing.	Supports a skin homing defect rather than systemic Treg deficiency/failure.	[21]
	Lili et al.	Human case–control study; flow cytometry and ex vivo assays	Activated IFN-γ-, granzyme B-, and perforin-positive CD8+ cells coexisted with defective Treg-mediated immune regulation.	Links defective regulation with enhanced melanocyte-directed effector activity.	[22]
	Miao et al.	Murine model; PD-L1 fusion protein	PD-L1 treatment restored about 60% of lost pigmentation, increased skin Tregs, and reduced melanocyte-reactive effector T cells.	Suggests that checkpoint reinforcement can reverse depigmentation.	[23]
	Ahmed et al.	Human skin cells and vitiligo tissue models	IFN-γ induced PD-L1 in healthy cells, whereas vitiligo melanocytes showed impaired PD-L1 upregulation.	Suggests melanocyte-intrinsic failure of protective checkpoint induction.	[24]
Axis 3. Regenerative capacity	Cui et al.	Human repigmenting lesions; histology and ultrastructure	Melanocytes were detected in the outer root sheath and perifollicular epidermis during repigmentation.	Identifies the hair follicle as the primary melanocyte reservoir for epidermal repigmentation.	[25]
	Nishimura et al.	Murine genetic models and follicular lineage analysis	Undifferentiated melanocyte-lineage cells localized to the permanent follicle; niche disruption altered their maintenance and differentiation.	Demonstrates dependence of melanocyte regeneration on a specialized follicular niche.	[26]
	Goldstein et al.	Human NB-UVB-treated skin; microdissection and transcriptomics	NB-UVB increased GLI1 in bulge precursors and activated β-catenin-associated programs involving proliferation, migration and differentiation of melanocytes.	NB-UVB promotes follicular melanocyte regeneration through β-catenin/GLI1 signaling	[27]
	Regazzetti et al.	Human biopsies, transcriptomics, stress experiments, and ex vivo treatment	WNT signaling was reduced in depigmented skin. Oxidative stress suppressed WNT, whereas WNT activation promoted premelanocyte differentiation ex vivo.	Identifies a potentially reversible block in precursor differentiation.	[28]
	Cao et al.	Stress-induced murine leukoderma/leukotrichia; single-cell RNA sequencing	Melanocyte populations showed distinct stress-related fates, and follicular compartments acquired IL-1, IL-6, IL-15, CCL2, CXCL12, and IFN-γ signatures.	Links regenerative failure to inflammatory remodeling of the follicular niche.	[29]
	Hamzavi et al.	Randomized within-subject trial of skin-cell suspension transplantation	Autologous cell suspension produced effective, durable repigmentation in selected stable lesions, with variable lesion-level outcomes.	Shows that cellular replacement can compensate for an insufficient endogenous reservoir.	[30]
Axis 4. Microenvironmental permissiveness	Wagner et al.	Human biopsies, reconstructed epidermis, and adhesion models	Melanocyte E-cadherin was reduced or discontinuous before visible depigmentation and associated with abnormal positioning and stress vulnerability.	Supports a pre-existing anchorage defect that reduces melanocyte retention.	[31]
	Gauthier et al.	Human skin samples; histopathology and ultrastructural analyses	Demonstrates melanocyte detachment and transepidermal elimination following minor mechanical stress.	Suggests that defective adhesion may contribute to melanocyte loss independently of immune-mediated destruction	[32]
	Komatsu et al.	Human iPSC-derived coculture and reconstructed skin; ROCK inhibition	IFN-γ impaired survival and dendrites. ROCK inhibition partially restored both and increased E-cadherin, DDR1, endothelin-1, and bFGF.	Shows that inflammatory adhesion and trophic defects are pharmacologically modifiable.	[33]

**Table 2:** Therapeutic interventions across the biological axes of repigmentation competence.

Axis	Intervention	Biological Rationale	Development Stage	Combination	Main Message	Ref
Axis 1	Ruxolitinib cream	JAK1/2 blockade	Approved	No	Validated IFN-γ/JAK targeting	[4,6,18]
	AMG-714	IL-15/TRM targeting	Phase 2 (completed-results posted)	No	Limited clinical translation	[34]
	Baricitinib	JAK1/2 inhibition	Clinical	No	Efficacy signal in vitiligo	[9]
	Upadacitinib	Selective JAK1 inhibition	Phase 2 (completed); Phase 3 ongoing	No	Efficacy in extensive NSV	[12,13]
	Ritlecitinib	JAK3/TEC inhibition	Phase 2b (completed); Phase 3 ongoing	No	Stabilization and repigmentation	[14,15,55]
	SHR0302/VC005	JAK 1 inhibition	Phase 2–3 (terminated by sponsor)	No	Emerging candidates	[16]
	VC005	JAK 1 inhibition	Phase 2 (recruiting)	No		[17]
Axis 2	MK-6194/PT101	Treg-selective IL-2 mutein	Phase 2a (terminated)	No	No efficacy data available	[44]
	Low-dose IL-2 approaches	Treg expansion	Translational	No	Strong rationale, unproven in vitiligo	[40,42,43]
Axis 3	NCES	Melanocyte replacement	Established	No	Durable repigmentation in selected lesions	[30]
Axis 3 + 1	Upadacitinib + NCES	Replacement + immune control	Ongoing	Yes	Explicit multi-axis strategy	[47]
Axis 3 + 4	NB-UVB	Niche activation	Established	No	Acts across multiple axes	[27]
Axis 4	Afamelanotide + NB-UVB	MC1R signaling	Clinical/ongoing	Yes	Enhanced repigmentation	[53,54,56]
Axis 1 + 3/4	Ruxolitinib + NB-UVB	Immune + regenerative engagement	Published/ongoing	Yes	Improved outcomes	[7,57]
	Baricitinib + phototherapy	Immune + regenerative engagement	RCT	Yes	Improved repigmentation	[10]
Axis 4	ROCK inhibition	Adhesion restoration	Preclinical	No	Proof-of-concept	[33]

## Data Availability

No new data were created or analyzed in this study. Data sharing is not applicable to this article.
